# What can an echocardiographer see in briefly presented stimuli? Perceptual expertise in dynamic search

**DOI:** 10.1186/s41235-020-00232-7

**Published:** 2020-07-21

**Authors:** A. J. Carrigan, P. Stoodley, F. Fernandez, M. W. Wiggins

**Affiliations:** 1 Centre for Elite Performance, Expertise and Training, Macquarie University, North Ryde, Australia; 2 Perception in Action Research Centre, Macquarie University, Blacktown, Australia; 3 Department of Psychology, Macquarie University, 4 First Walk, North Ryde, NSW 2109 Australia; 4 School of Medicine, Western Sydney University, Blacktown, Australia; 5 Westmead Private Cardiology, Westmead, Australia; 6 Blacktown Mount Druitt Hospital, Sydney, Australia

**Keywords:** Echocardiography, Vision, Perception, Expertise

## Abstract

**Background:**

Experts in medical image perception are able to detect abnormalities rapidly from medical images. This ability is likely due to enhanced pattern recognition on a global scale. However, the bulk of research in this domain has focused on static rather than dynamic images, so it remains unclear what level of information that can be extracted from these displays. This study was designed to examine the visual capabilities of echocardiographers—practitioners who provide information regarding cardiac integrity and functionality. In three experiments, echocardiographers and naïve participants completed an abnormality detection task that comprised movies presented on a range of durations, where half were abnormal. This was followed by an abnormality categorization task.

**Results:**

Across all durations, the results showed that performance was high for detection, but less so for categorization, indicating that categorization was a more challenging task. Not surprisingly, echocardiographers outperformed naïve participants.

**Conclusions:**

Together, this suggests that echocardiographers have a finely tuned capability for cardiac dysfunction, and a great deal of visual information can be extracted during a global assessment, within a brief glance. No relationship was evident between experience and performance which suggests that other factors such as individual differences need to be considered for future studies.

## Significance

Decades of research in the medical image perception field has demonstrated that with exposure, practitioners develop a type of perceptual fine tuning which allows for the efficient and accurate diagnosis within a medical image. However, the bulk of the research has focused in radiological domains presenting static stimuli (e.g. mammograms) and little is known about the visual processing of dynamic medical stimuli such as real-time imaging (e.g. ultrasound). This study presents three experiments that investigate expertise in dynamic medical imaging by presenting dynamic stimuli (echocardiograms) to specialist practitioners (echocardiographers). The participants viewed echocardiograms to investigate expertise in visual processing. Their tasks were to first detect an abnormality in cardiac function and then to subsequently categorize the level of dysfunction. As predicted, the echocardiographers were above chance on both tasks. However, for the categorization errors, the participants responded with the more abnormal category, adopting a liberal criterion for disease severity. This is significant, as it implies that targeting training may be necessary to improve sensitivity on categorization. The results were not related to level of experience, which suggests that other factors are involved in the development of expertise in echocardiography such as individual differences. These findings have important implications: currently within the profession, once a level of proficiency is reached, often there is no ongoing feedback or support provided. Although the echocardiographers were able to extract a large amount of information in a brief glance, targeted training with ongoing feedback may reduce error.

## Background

Echocardiographers have an important role in diagnostic medicine: they perform echocardiograms—the most common, non-invasive, imaging technique in cardiology (Lang et al. 2015). During an echocardiogram, the echocardiographer operates an ultrasound machine, which transmits sound waves via a transducer held against a patient’s thorax. The sound waves, directed towards and reflected from the heart, are used to generate images that are displayed on a screen in real time. These images are stored for future analysis by a physician.

Measurement and analysis of images most often occurs during the course of the echocardiogram. To do so, echocardiographers must first acquire and visually search images, identify and capture normal and abnormal features, and perform complex anatomical and hemodynamic measurements. Often, as many as 80 images (a combination of still and moving images, with and without measurements) are stored in the course of a routine 30-min exam. As such, the echocardiographer’s image acquisition, visual search of the display, measurement of features, and diagnostic decisions must occur quickly. Searching for abnormalities and formulating diagnostic decisions are perceptually and cognitively demanding which means that the potential for error can be high.

The visual search errors to which humans are prone become particularly problematic in high consequence environments such as diagnostic medicine. Errors that can occur in the context of diagnosis include missing targets that are present (false negatives), or false alarms on target-absent displays (false positives). False negatives, in particular, result in missed abnormalities, which can have significant consequences for patients. For echocardiographers, a missed ventricular wall motion abnormality in an echocardiogram before routine surgery may be indicative of significant coronary artery disease with implications for survival through anesthesia.

Michelena et al. (2013) reported that echocardiography errors in the measurement of aortic stenosis, a serious valvular disease requiring surgery, occurred in one-third of cases. Within a pediatric echocardiography setting, Benavidez et al. (2014) reported that 70% of diagnostic error cases were false negatives, 15% false positives, and 15% discrepant diagnoses. Cognitive errors accounted for 37% of total diagnostic error. The factors contributing to error included misidentification/interpretation of a finding, under interpretation/overinterpretation of a finding and distraction by another diagnosis (Benavidez et al. 2008). In radiology, where the bulk of medical image perception research has occurred, there is a reported miss error rate of 30%, with an equally high rate of false alarms (Berlin 2005). Importantly, approximately 60% of errors can be attributed to cognitive or perceptual factors (Brem et al. 2003). Using eye tracking, radiologists’ errors were categorized when reading chest radiographs into three main categories: visual search errors; where they never fixate the abnormality (30%); recognition errors, where the abnormality is fixated but only briefly (25%); and decision errors, where the abnormality is fixated but actively dismissed as an abnormality (45%) (Kundel et al. 1978). Carrigan et al. (2015) studied the visual search behavior of ultrasound technologists (who perform general, not cardiac, scans) and showed that they were prone to decision errors. These studies show that errors clearly occur in diagnostic medicine; the cost of these errors in both financial and social terms makes it crucial to examine the cognitive processes underpinning visual search in medical imaging.

Evidence from the natural scene literature has demonstrated that a large amount of information is processed in the first glance at a visual scene (Carrigan et al. 2019b; Fei-Fei et al. 2007; Potter 1976; Potter et al. 2010; Thorpe et al. 1996; VanRullen and Thorpe 2001). An exposure duration of 100 ms is sufficient for observers to extract the basic meaning of natural scenes (e.g. indoor versus outdoor (Potter 1976)). It is widely accepted that rapid scene categorization is based on a global summary or “gist” (Oliva 2005). Described as the earliest meaningful stage of scene perception, after or during a glance, gist captures the global properties and overall spatial layout of a scene (Torralba et al. 2010). These properties are based on statistical and structural cues in the scenes and stimulus-based information such as the low-level features within the scene. People tend to extract low-level visual information such as size, motion, and orientation rapidly (Greene and Oliva 2009; Hidalgo-Sotelo et al. 2006; Oliva and Torralba 2001; Wolfe et al. 2011). It has been proposed that the ability to extract information rapidly from a scene is the result of experience with the environment (Drew et al. 2013). This “expertise” means that visual perceptual skills after years of interacting with the surrounds undergoes fine tuning, which supports the rapid processing of scenes.

Those with expertise in a particular domain can also rapidly extract a large amount of relevant information from features in the environment (Abernethy 1987; Kundel and Nodine 1975; Nodine and Krupinski 1998), where a superior ability develops to encode large scale visual patterns (Drew et al. 2013). In the context of medical imaging, radiologists, but not naïve participants, can detect abnormalities at above what is expected by chance after viewing the images for < 1 s (Brennan et al. 2018; Carrigan et al. 2019a; Donovan and Litchfield 2013; Evans et al. 2013; Evans et al. 2016; Kundel and Nodine 1975; Kundel et al. 2008). Eye-tracking studies indicate that expert radiologists fixate faster and more accurately on an abnormality in mammographic images than less-experienced radiologists and use fewer eye movements to do so (Kundel and La Follette Jr 1972; Kundel and Nodine 1975). For example, within 300 ms, on average, mammographic readers fixate on 67% of breast cancers (Kundel et al. 2008). Of course, presenting images briefly is not the typical way radiologists read images in clinical practice. There are other image projections, previous imaging and clinical history available to a reporting radiologist who would conduct a review under free-viewing conditions. However, there is some evidence that what is processed in the first second influences the overall diagnosis (Mello-Thoms 2009). Understanding this ability is critical as important decisions (e.g. medical diagnosis) often depend on this early processing.

Historically, the bulk of the literature in medical image perception has focused on performance interpreting static images. However, there is evidence that a similar pattern to what has been reported with static images exists for dynamic images. Experts are more accurate and are faster to fixate and attend to more relevant features of complex dynamic stimuli than novices. These findings have been reported in diverse domains such as billiards (Crespi et al. 2012), fish locomotion (Jarodzka et al. 2010), closed caption television (Howard et al. 2013), as well as medicine (Balslev et al. 2012; Wu et al. 2019). Loveday et al. (2013) studied pediatricians and novices interpreting both static and dynamic stimuli (patient bedside monitors). They showed that in the absence of dynamic cues, the experts maintained performance, whereas the novices did not.

In the radiological domain, radiologists scrolling through computer tomographic (CT) images or digital breast tomograms (DBT) may use motion cues when interpreting volumetric scans. For example, nodules may capture attention as they flicker in and out of view as the radiologist navigates though the scan (Williams and Drew 2019). Wu et al. (2019) presented radiologists a series of DBT scans presented for 1.5 s. They showed that the participants’ accuracy was comparable to briefly presented static mammography. These findings suggest that experts can utilize a global signal from dynamic cues when making a diagnostic decision.

One of the most common diagnostic assessments routinely performed by echocardiographers is a global and regional evaluation of the left ventricular ejection fraction (LVEF). This parameter is used to assess systolic function of the left ventricle (LV) which predicts the prognosis of patients with disease such as coronary artery disease and congestive heart failure. An accurate assessment is critical as the outcome guides therapeutic decisions. Using echocardiography is advantageous due to its non-invasive nature and relatively low cost. Moreover, the portable machine allows the examination to be performed in critical care. It is safe and the results are instantaneous (Shahgaldi et al. 2009).

The LV examination can be performed quantitatively using validated, real-time, three-dimensional measurements such as biplane Simpson, and quantitatively with a visual assessment by the operator, termed the eyeball method. In practice, as the quantitative method is time-consuming, the eyeball method is the preferred method and is routinely used as results can be rapidly obtained (Gudmundsson et al. 2005).

Shahgaldi et al. (2009) compared the qualitative and quantitative methods in the assessment of systolic LV function on 30 cases and showed that these two methods were highly correlated between observers (*r* = 0.91–0.95). However, a limitation of this study was that they only included two experienced echocardiographers as observers, which may not capture the true variability present in clinical practice. Indeed, the eyeball method would be dependent on the skill of the echocardiographer, so it is plausible that the methods are more discrepant than what have been reported.

The overall goal of the current study was to investigate the diagnostic performance of echocardiographers and explore the level of information available after a brief exposure to a dynamic stimulus. Specifically, the information was extracted using a qualitative assessment of cardiac function. This was achieved by conducting three experiments with three groups of echocardiographers and one group of naïve participants. In Experiment 1, we presented 3s movies, where the tasks were the visual detection and the categorization of ventricular contractile function. Experiment 2, was identical except *a priori* we conducted an independent verification of the abnormal category image labels, reduced the movie presentation time to 2 s, and obtained data from echocardiographers and naïve participants. To our knowledge, the detection sensitivity on LV dysfunction in the clinic with free viewing has not been quantified. However, it was estimated by two subject matter experts that 10 s would be long enough to observe whether the LV was normal and to subsequently categorize it. Thus, to establish a baseline of performance and emulate “real-world” practice, in Experiment 3, the movies were presented to a new group of echocardiographers for either 1 s or 10 s.

It was hypothesized that: (1) echocardiographers would demonstrate high levels of ventricular dysfunction detection and categorization accuracy after seeing a dynamic image briefly, compared with naïve participants; and (2) self-reported years of experience practicing as an echocardiographer would be related to accuracy.

## Experiment 1: Diagnostic performance of echocardiographers viewing 3s stimuli

### Method

#### Participants

Data were collected from 44 echocardiographers who volunteered in a teaching or a conference setting. The majority of the sample were female (81%), which is slightly higher when compared to the distribution of females within the echocardiography population within Australia (71%).

The sample consisted of six students, three of whom were in the first year of their training and three in their second year. The mean self-reported years of experience for both students and qualified echocardiographers was 13 years (standard deviation [SD] = 9, range = 1–35 years). The mean number of cases performed per week was 24 (SD = 11), the mean number of cases per year was 990 (SD = 680), and 88% of the participants were accredited with a governing professional board (ASAR). All but one of the participants was right-handed, all reported normal or corrected-to-normal vision, and all were naïve to the purposes of the experiment. In return for participation, they were offered the opportunity to win an iPad.

#### Demographic survey

The participants were asked to indicate their age, sex, handedness, whether they were accredited with the ASAR, self-reported number of years of experience in echocardiography, the number of cases performed per week, and the number of cases performed per year.

#### Diagnostic performance

**Fig. 1:**
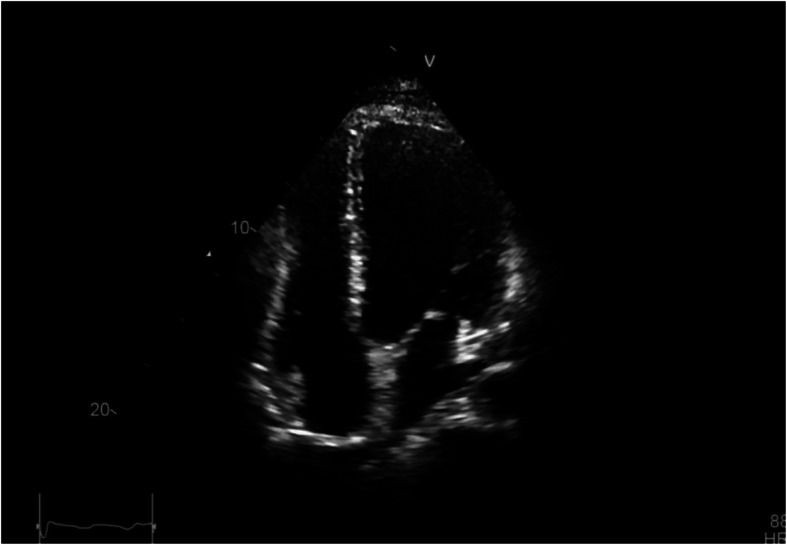
Exemplar of an apical four-chamber cardiac still image from the stimuli set

Diagnostic performance was assessed using detection and categorization tasks. The stimuli consisted of 84 movies of the heart. All 84 de-identified images had been acquired from a single imaging plane (the apical four-chamber view), were from a teaching set belonging to two of the authors (PS and FF), and were converted from DICOM to MP4 format for display (see Fig. 1). Two exemplar movies from the normal and severe category can be found at https://osf.io/vez4w/?view_only=82a630399de54e8fae05c51f45675c97. Participants were asked to qualitatively evaluate left ventricular (LV) contractile function by estimating the LVEF—the most common method used for this purpose.

The target present stimuli (*n* = 42) consisted of movies showing varying degrees of LV dysfunction: mild dysfunction (LVEF in the range of 41%–51%; *n* = 14), moderate dysfunction (LVEF 30%–40%; *n* = 14), and severe dysfunction (LVEF < 30%; *n* = 14). The target absent stimuli (*n* = 42) consisted of movies showing normal function (LVEF > 52%). The frame of each unique movie was covered with a black mask to remove all the distracting information such as machine characteristics. The central fixation point was a cross measuring 0.5° of visual angle which appeared against a black background (RGB triplet: 0,0,0).

The stimuli were presented on a Gigabyte P55W, full high definition (HD), 15-in. laptop, resolution 1600 × 900 pixels, refresh rate 60 Hz, and presented using MATLAB via PsychToolbox 3 (Kleiner et al. 2007). Stimuli were downsized to 636 (width) × 434,444 or 476 (height), looped, and displayed for 3 s.

#### Procedure

The study was approved by the institutional review board at Macquarie University and informed consent was obtained for each participant. The experiment was conducted in a room either in a teaching or a conference setting. After completing a series of demographic questions, the detection and categorization tasks commenced with six practice trials (50% abnormal) using movies not part of the main experiment, followed by 84 experimental trials (50% abnormal). Each trial started with a 100ms flash of a central fixation cross and subsequent fixation for 500 ms, followed by a centrally presented apical four-chamber view of the movie looped approximately 2–3 times for 3 s. Depending on the patient’s heart rate, which varied slightly dependent on patient’s age, sex and general health, each movie presented 2–3 cardiac cycles.

**Fig. 2:**
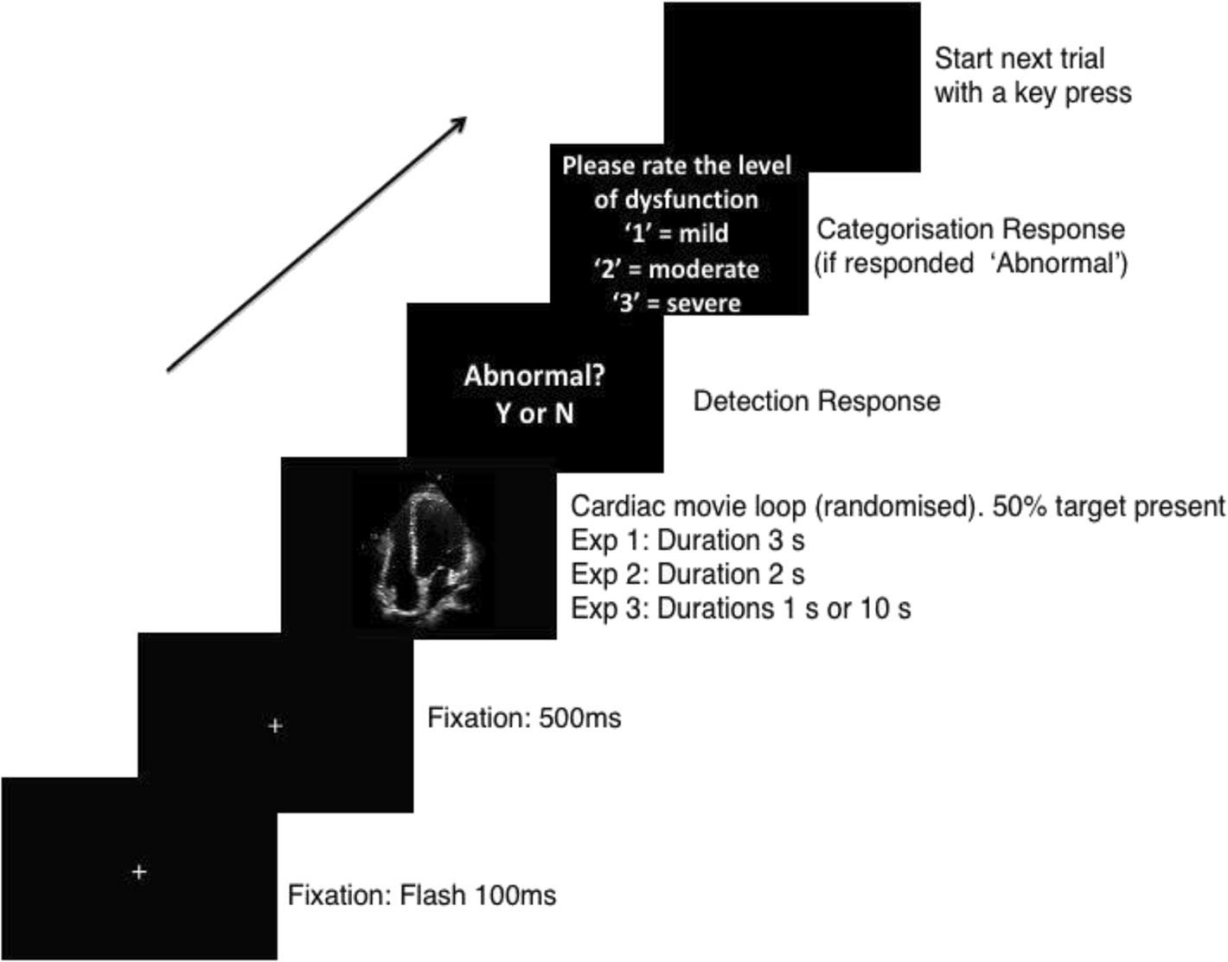
Example of an experimental trial for diagnostic performance shown to the participants in three experiments. Trials began with a fixation cross followed by the cardiac movie and a response screen for detection. The subsequent categorization screen was displayed if the participant responded “Yes” for abnormal. Note: Movie durations were as follows: Experiment 1: 3 s, Experiment 2: 2 s, Experiment 3: Either 1 s or 10 s

After each movie, the participants were presented a black screen asking them to respond to whether the movie was either “abnormal”? (yes: “Y”; no: “N”) with a key press. If they selected “yes,” they were presented with a subsequent screen that asked them to categorize the severity of dysfunction: “1” = mild; “2” = moderate; and “3” = severe. If they selected “no,” they were prompted to begin the next trial. Trials timed out after 6 s. The echocardiographers commenced the next trial with a key press and no feedback was provided (see Fig. 2).

### Results

Statistical analysis was performed using IBM Statistical Software for the Social Sciences (SPSS Version 25) and the open source software package JASP (JASP team 2016). For each test both frequentist statistics and Bayes Factors (BF_10_) with a Cauchy prior width of 0.707 are reported. A BF < 1 indicates that the data support the null rather than the alternative hypothesis, a BF 1–3 indicates *anecdotal* or *weak* support for the alternative hypothesis, whereas a BF > 3 suggests *strong* evidence for the alternative and a BF > 10 suggests *very strong* evidence for the alternative (Kass and Raftery 1995). The dependent variables were accuracy (% correct) and sensitivity (*d′)*.

#### Diagnostic performance

The first hypothesis was that echocardiographers would demonstrate high levels of accuracy after seeing a dynamic movie briefly.

##### Ventricular dysfunction detection

Accuracy was calculated as the percentage of correct trials of the total trials seen (*n* = 84). Sensitivity was measured using *d′*, a measure that considers an observer’s hits (responding abnormal when abnormal) and their false alarms (responding abnormal when normal) (see Table 1). A *d′* of zero indicates that participants are performing at chance (i.e. no better than guessing). A single sample t-test on mean *d′* (2.26) relative to chance (0), showed that the echocardiographers were able to accurately detect normal and abnormal ventricular function above chance; *t* (43) = 33.06, *p* < 0.0001, BF_10_ = 6.561e + 28.

**Table 1:** Experiment 1: Accuracy and sensitivity for the detection task for the echocardiographers viewing each movie for 3 s (*n* = 44)

Dependent measure	Mean	SD
Total correct (%)	81.92	7.5
Target present correct (%)	94.21	4.8
Target absent correct (%)	69.64	17.07
Sensitivity (*d′*)	2.26	0.45

##### Ventricular dysfunction categorization

Accuracy for each of the categories (mild, moderate, severe) was calculated as the proportion of number correct to the total detection correct from each category (see Table 2). A single samples t-test on mean dysfunction correct relative to chance (33.33%) showed that the echocardiographers were able to accurately categorize abnormal ventricular function above chance; *t* (43) = 37.6, *p* < 0.0001, BF_10_ = 1.219e + 31.

**Table 2:** Experiment 1: Mean percentage accuracy for the ventricular function categorization task when detection was correct (*n* = 44)

Category	Mean (% correct)	SD (% correct)
Total dysfunction	61.84	5.03
Mild	41.32	16.53
Moderate	47.21	11.54
Severe	90.87	13.85

For the errors, across all the correct target present movies, the echocardiographers incorrectly responded “severe” 62.92% of the time, “moderate” 32.46%, and “mild” 4.6%, where the actual prevalence was 33.33%. This suggests that the echocardiographers show a bias towards responding with “severe.” Across all of the echocardiographers, the majority of the false alarms (saying abnormal on normal cases) were incorrectly categorized as mild (M = 80.76%, SD = 19.72), then moderate (M = 14.71%, SD = 16.01), and severe (M = 1.98%, SD = 5.69). The proportion of trial timeouts for the false alarms was 2.27% (SD = 5.6).

Consistent with the hypothesis, we tested whether self-reported years of experience correlated with accuracy on diagnostic performance. There was no statistically significant correlation evident for years of experience and ventricular dysfunction accuracy (Pearson’s *r* (44) = 0.19, *p* = 0.22, BF_10_ = 0.39). However, for dysfunction categorization accuracy there was a significant, small, positive correlation between years of experience and the echocardiographers’ ability to categorize ventricular dysfunction (Pearson’s *r* (44) = 0.3, *p* = 0.486, BF_10_ = 1.19). Note: A BF_10_ 1–3 indicates *anecdotal* or *weak* support for the alternative hypothesis. There were no other statistically significant correlations evident (*p* > 0.05).

### Discussion

Experiment 1 investigated the diagnostic accuracy of echocardiographers performing a domain-specific task. The first aim was to examine accuracy for detection and categorization of ventricular function after the brief presentation of 84, 3s movies using a qualitative assessment. The results indicated that the echocardiographers were highly accurate (81.92%) in detecting an abnormality, with sensitivity above chance (*d′* = 2.26). This provides evidence to suggest that, like radiologists, echocardiographers are able to detect abnormalities after brief presentations of task-relevant stimuli. This finding was not surprising as the assessment LV contractile function is an integral part of most routine echocardiograms; therefore, the participants would be familiar with this diagnostic task. Moreover, the selected movie duration of 3 s in the study may have been too long, making the distinction between normal and abnormal an overly straightforward task, thereby not capturing this aspect of visual expertise precisely.

For the categorization of cardiac ventricular dysfunction (when the initial review revealed an abnormality), the results indicated that the overall accuracy was not high (61.84%), especially when differentiating between mild and moderate dysfunction. A plausible explanation for these results is that the stimuli may not have been accurately represented by the labelled categories. That is, stimuli with LVEF values at the upper or lower limits of a category, or an LVEF of 42% in the mild (41%–51%) category, for example, may have made differentiation particularly difficult. To investigate whether the stimuli were categorized accurately, two independent, experienced echocardiographers who were blind to the purposes of the task, verified the stimuli. These results revealed two discrepant images with respect to category (one mild, one moderate).

To address these issues, a follow-up experiment was designed with the following modifications: (1) duration of movie presentation was reduced to 2 s; (2) the discrepant movies were switched into the correct categories; and (3) a new sample of echocardiographers and a comparison group of naïve participants were recruited to examine whether echocardiographers have finely tuned perceptual capabilities for clinically relevant stimuli.

## Experiment 2: Diagnostic performance of echocardiographers and naive participants viewing 2-s stimuli

### Method

#### Echocardiographers

Data were collected from 30 echocardiographers who volunteered in a conference setting. Eighteen participants (60%) were female, all but one of whom was qualified and board-accredited and the other was a trainee. Mean self-reported years of experience for both the student and qualified echocardiographers was 12 years (SD = 10, range = 1–39 years). The mean number of cases performed per week was 38 (SD = 16) and the mean number of cases per year was 1622 (SD = 813). All but four of the participants were right-handed, all reported normal or corrected-to-normal vision, and all were naïve to the purposes of the experiment. In return for participation, they were offered the chance to win an iPad.

#### Demographic survey

The echocardiographers were asked to indicate their age, sex, handedness, number of years of experience in echocardiography, and number of cases performed per week and per year. They were also asked whether they were an accredited sonographer with the ASAR and their workplace environment (public hospital, private hospital, private practice, or a combination). Two participants did not respond to the demographic survey.

#### Diagnostic performance and procedure

Diagnostic performance was assessed using the identical detection and categorization tasks that were presented in Experiment 1, except the video presentation duration was reduced to 2 s (1–2 movie loops). The procedure was identical to Experiment 1.

#### Naïve participants

The naïve participants comprised 30 students from the Macquarie University undergraduate community (17 female; median age = 22 years, SD = 11 years, range = 17–70 years) who participated in exchange for course credit. Five were right- handed; all reported normal or corrected-to-normal vision and were naïve to the purposes of the experiment. All reported no experience with medical images, specifically echocardiograms. The stimuli were presented on a DELL 15-in., full HD laptop, resolution 1600 × 900 pixels, refresh rate 60 Hz, and presented using MATLAB via PsychToolbox 3 (Kleiner et al. 2007). Stimuli were downsized to 636 (width) × 434,444 or 476 (height), looped and displayed for 2 s.

#### Demographic survey

The naïve participants were asked to indicate their age, sex, and handedness.

#### Diagnostic performance and procedure

Diagnostic performance was assessed using the identical detection and categorization tasks and procedure that were presented to the echocardiographers.

### Results: Echocardiographers

A series of correlations were conducted between the demographic variables and accuracy on the diagnostic task for 30 participants. For detection and categorization, there were no statistically significant correlations evident that related to accuracy (*p* > 0.05).

#### Diagnostic performance

##### Ventricular dysfunction detection

Accuracy was calculated as the percentage of correct trials of the total trials seen (*n* = 84). Sensitivity (*d* prime) was calculated as reported in Experiment 1 (see Table 3). A single sample *t*-test on mean *d′* (0.21) relative to chance (0), showed that the echocardiographers were able to accurately detect normal and abnormal ventricular function above chance; *t* (29) = 30.86, *p* < 0.0001, BF_10_ = 2.955e + 20.

**Table 3:** Experiment 2: Accuracy and sensitivity for the detection task for the echocardiographers viewing each movie for 2 s (*n* = 30)

Dependent measure	Mean	SD
Total correct (%)	82.62	5.57
Target present correct (%)	92.31	7.55
Target absent correct (%)	72.94	11.99
Sensitivity (*d′*)	2.2	0.39

##### Ventricular dysfunction categorization

Accuracy for each of the categories (mild, moderate, severe) was calculated as the proportion of correct targets against the total number of targets present trials from each category (see Table 4).

**Table 4:** Experiment 2: Mean percentage accuracy for the ventricular function categorization task when detection was correct for the echocardiographers (*n* = 30)

Category	Mean (% correct)	SD (% correct)
Total dysfunction	63.12	9.33
Mild	45.47	16.53
Moderate	51.35	16.74
Severe	90.1	12.1

A single sample t-test comparing mean dysfunction correct relative to chance (33.33%) showed that the echocardiographers were able to accurately categorize abnormal ventricular function above chance; *t* (29) = 17.48, *p* < 0.0001, BF_10_ = 8.50e + 13.

As for Experiment 1, a consistent pattern was evident for the categorization errors when detection was correct: “severe” = 59.15%; “moderate” = 34.81%; and “mild” = 6.04%, where actual prevalence = 33.33%. This again suggests that when the echocardiographers are uncertain, they responded with a “severe” classification. The majority of the false alarms in Experiment 2 were also incorrectly categorized as mild (M = 77.02%, SD = 20.88), then moderate (M = 16.52%, SD = 21.12), and severe (M = 0.64%, SD = 2.55). The proportion of trial timeouts for the false alarms was 6.02% (SD = 9.52).

Experiment 2 tested whether self-reported years of experience correlated with accuracy on diagnostic performance. There were no statistically significant correlations evident for years of experience and ventricular dysfunction detection accuracy (Pearson’s *r* (28) = −0.01, *p* = 0.95, BF_10_ = 0.23) or ventricular dysfunction categorization accuracy (Pearson’s *r* (28) = 0.05, *p* = 0.82, BF_10_ = 0.24). There were no other statistically significant correlations evident (*p* > 0.05).

#### Results: Naïve participants

A series of correlations were conducted between the demographic variables and accuracy on the diagnostic task for 30 participants. For detection there was a significant, negative correlation between age and target absent accuracy (*r* = −0.44, *p* = 0.015). There were no other statistically significant correlations evident that related to accuracy (*p* > 0.05).

#### Diagnostic performance

##### Ventricular dysfunction detection

Accuracy was calculated as the percentage of correct trials of the total trials seen (*n* = 84). Sensitivity (*d* prime) was calculated as reported in Experiments 1 and 2 (see Table 5). A single sample *t*-test on mean *d′* (0.21) relative to chance (0) showed that the naïve participants were above chance on discrimination between normal and abnormal ventricular function; *t* (29) = 2.44, *p* = 0.02, BF_10_ = 2.44.

**Table 5:** Experiment 2: Accuracy and sensitivity for the detection task for the naïve participants viewing each movie for 2 s (*n* = 30)

Dependent measure	Mean	SD
Total correct (%)	53.85	8.05
Target present correct (%)	54.84	15.09
Target absent correct (%)	52.86	18.59
Sensitivity (*d′*)	.21	.46

##### Ventricular dysfunction categorization

Accuracy for each of the categories (mild, moderate, severe) was calculated as the proportion of correct targets against the total number of targets present trials from each category (see Table 6).

**Table 6:** Experiment 2: Mean percentage accuracy for the ventricular function categorization task when detection was correct for the naive participants (*n* = 30)

Category	Mean (% correct)	SD (% correct)
Total dysfunction	32.45	11.07
Mild	31.82	17.98
Moderate	45.22	53.10
Severe	27.97	18.94

A single sample t-test comparing mean dysfunction correct relative to chance (33.33%) showed that the naïve participants were not able to accurately categorize abnormal ventricular function above chance; *t* (29) = −0.43, *p* = 0.67, BF_10_ = 0.21. For the categorization errors when detection was correct, the naïve participants responded: “severe” = 41.43%; “moderate” = 38.67%; and “mild” = 19.91%, where actual prevalence = 33.33%. The majority of the false alarms were incorrectly categorized as mild (M = 41.79%, SD = 18.87), then moderate (M = 34.57%, SD = 11.33), and severe (M = 22.82%, SD = 14.16). The proportion of trial time outs for the false alarms was 1.38% (SD = 4.82).

To test the hypothesis that echocardiographers have medical image expertise for these stimuli, an independent samples t-test between the echocardiographers and the naïve participants on D prime was performed and showed that the echocardiographers were significantly more accurate on the detection task compared with the naïve participants; *t* (29) = 18.92, *p* < 0.00001. BF = 6.533e + 14. A second t-test on dysfunction rating accuracy also confirmed that the echocardiographers were also significantly more accurate on the categorization task, compared with the naïve participants; *t* (29) = 12.89, *p* < 0.00001, BF = 4.641e + 10.

### Discussion

Experiment 2 showed that even though the naïve participants were above chance (50%) for detection, but not categorization, performance was lower compared with echocardiographers. This result is not surprising and suggests that echocardiographers share perceptual fine tuning for relevant diagnostic features. However, due to the small amount of research with echocardiographers reported in the literature, we do not yet know how echocardiographers would perform when the stimuli are very brief, or what detection or categorization sensitivity for LV dysfunction is in clinical practice. Thus, Experiment 3 was designed to examine these factors.

## Experiment 3: Diagnostic performance of echocardiographers viewing 1s and 10s stimuli

Experiment 3 was designed with the following aims: (1) to examine whether experts are able to extract information from very brief stimuli; and (2) to examine performance at a longer duration, in keeping with clinical practice, experimentally and subjectively. Data were collected from a group of echocardiographers presented with two movie durations—brief (1 s) and extended (10 s)—to provide information about performance on a range of presentation durations. The participants were also asked to complete a brief questionnaire about their perceived performance in practice.

### Method

Data were collected from 14 qualified echocardiographers who volunteered in their workplace during their breaks. Eight (57%) were female. Mean self-reported years of experience for the echocardiographers was 14.35 years (SD = 10.53, range = 3–38 years). The mean number of cases performed per week was 45 (SD = 17) and the mean number of cases per year was 2539 (SD = 3043). Eight echocardiographers worked in a private setting, four in a public hospital, and three in both settings. All but three of the participants were right-handed, all reported normal or corrected-to-normal vision, and all were naïve to the purposes of the experiment.

#### Demographic survey

The echocardiographers were asked to indicate their age, sex, handedness, number of years of experience in echocardiography, and number of cases performed per week and per year. They were also asked whether they were an accredited sonographer with the ASAR and their workplace environment (public hospital, private hospital, private practice, or a combination). To understand the typical qualitative, clinical assessment of LV systolic function, additional information was included: (1) “How long, on average, would you assess systolic LV function from the four-chamber view?” (2) “How long, on average, would it take you to detect whether the LV systolic function is normal, or not? (seconds and cardiac cycles);” and (3) “How long, on average, would it take you to subsequently categorize the level of dysfunction (mild/moderate/severe)?”

#### Diagnostic performance and procedure

Diagnostic performance was assessed using the identical detection and categorization tasks that were presented in Experiments 1 and 2, except the movie presentation duration varied across two groups of participants. Two subject matter experts and co-authors estimated 10 s to be adequate for the detection and categorization decision in practice; therefore, two durations were presented: Group 1 (*n* = 7), observed the movies for 1 s (~ 1 loop); and Group 2 (*n* = 7) for 10 s (~ 10–12 loops). The participants were randomly assigned to the two duration groups. The procedure was identical to Experiments 1 and 2.

### Results

Due to the sample size in each group, only the descriptive statistics are reported.

#### Demographic survey

The responses for the clinical assessment component of the survey are presented in Table 7.

**Table 7:** Subjective clinical assessment of left ventricular (LV) systolic function reported by 14 echocardiographers

Echocardiographer	Length of assessment of LV function (s)	Detection of LV abnormality (s) /(cardiac cycles)	Categorization of level of LV dysfunction (s)
1	1800	3/2	3
2	3	3/2–3	3–5
3	3	5/4	5
4	1800	10/10	30
5	7	7/4	10
6	5	3/3	15
7	10	10/10	20
8	5	5/5	10
9	10	5/2	20
10	5	5/3	5
11	60	15/2	30
12	3	3/3	3
13	3	3/3	3
14	2	2/2	3

#### Diagnostic performance

##### Ventricular dysfunction detection

Accuracy was calculated as the percentage of correct trials of the total trials seen (*n* = 84). Sensitivity (*d* prime) was calculated as reported in Experiments 1 and 2 (see Table 8).

**Table 8:** Experiment 3: Mean percentage accuracy and sensitivity for the detection task (*n* = 14)

Duration	% Total correct (SD)	% Target present correct (SD)	% Target absent correct (SD)	Sensitivity (d′)
1 s (*n* = 7)	88.43 (3.13)	87.41 (8.44)	89.46 (7.76)	2.57
10 s (*n* = 7)	86.39 (5.79)	95.23 (2.75)	77.55 (13.67)	2.54

##### Ventricular dysfunction categorization

Accuracy for each of the categories (mild, moderate, severe) was calculated as the proportion of correct targets against the total number of targets present trials from each category (see Table 9).

**Table 9:** Experiment 3: Mean percentage accuracy for the ventricular function categorization task when detection was correct (*n* = 14)

Duration	% Total dysfunction (SD)	% Mild (SD)	% Moderate (SD)	% Severe (SD)
1 s	58.88 (10.75)	56.3 (19.81)	57.15 (22.09)	71.43 (22.59)
10 s	61.75 (5.21)	30.77 (8.61)	67.53 (21.55)	97.96 (3.49)

For the categorization errors when detection was correct for the 1s movie duration condition, the echocardiographers responded: “moderate” = 44.44%; “severe” = 37.88%; and then “mild” = 17.68%. For the 10s movie duration, the echocardiographers responded: “severe” = 67.05%, “moderate” = 33.33%, and then “mild” = 1.89%, where actual prevalence = 33.33%. The majority of the false alarms in Experiment 3 were also incorrectly categorized as mild (mean 1 s = 72.29%, SD = 24.85; mean 10 s = 81.15%, SD = 21.07), then moderate (mean 1 s = 22.84%, SD = 22.87; mean 10 s = 15.52%, SD = 21.9), and last severe (mean 1 s = 0.0%, SD = 0; mean 10 s = 0.95%, SD = 2.52). There were no trial timeouts on the false alarm trials.

**Fig. 3:**
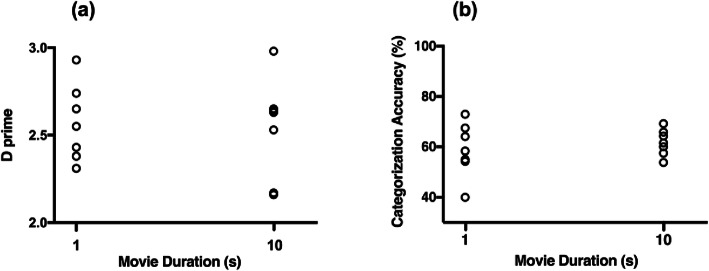
Performance on the left ventricular function assessment task for 14 echocardiographers. Seven participants observed the movies for 1 s and seven participants for 10 s. Panel (**a**) illustrates D prime and (**b**) categorization accuracy. Each point represents an individual echocardiographers’ score

The raw data for the echocardiographers on both detection and categorization for the two movie durations are presented in Fig. 3.

In Fig. 3, although there is more variance on D Prime scores, especially at the longer duration, overall these data suggest that viewing the stimuli for longer does not offer a performance advantage over the shorter duration. This suggests that echocardiographers are able to qualitatively detect and, to some degree categorize, ventricular dysfunction in a briefly presented echocardiogram.

### General discussion

Successful screening and interpretation of medical images is crucial in diagnostic medicine. To our knowledge, this is the first study that has investigated the diagnostic performance of a group of echocardiographers whose role is to perceive and interpret echocardiograms. Across three experiments that investigated echocardiographers’ and naïve participants’ qualitative assessment of cardiac function, we showed that the echocardiographers were able to accurately detect abnormal ventricular wall motion and to a lesser degree, subsequently categorize the level of dysfunction, after briefly presented echocardiograms.

Experiment 2 showed that the naïve participants were above chance (50%) for detection, but not categorization. This result may reflect the salience of the abnormal motion of the ventricle compared with the other cardiac chambers, even for the untrained observer. The more difficult task of categorization was not above chance. Importantly, the echocardiographers were more accurate than the naïve observers on both tasks. This suggests that even for dynamic stimuli experts can extract visual information rapidly.

These findings are consistent and extend what is known about medical image perceptual expertise. Decades of medical image perception research has shown that experts can accurately identify a static stimulus with an abnormality (Brennan et al. 2018; Carrigan et al. 2018; Evans et al. 2013; Kundel and Nodine 1975).

Researchers in vision science have shown that observers tend to rapidly extract low-level visual information such as motion from a scene (Greene and Oliva 2009; Hidalgo-Sotelo et al. 2006; Oliva and Torralba 2001; Wolfe et al. 2011). For an echocardiographer, motion cues present in dynamic scans (e.g. cardiac rhythm/wall motion) are crucial and provide the necessary information for a diagnosis.

As the echocardiographers were requested to make a judgment based on cardiac dynamics, we deemed that the movie in our study needed to be sufficiently long to allow for the extraction of diagnostic information, yet short enough to measure early visual processing. In studies investigating radiologists’ early visual processing, to capture the element of visual expertise, the presentation durations of static images are very brief (e.g. 250 ms; Carrigan et al. 2018). The duration required for dynamic stimuli to capture this element of expertise is unclear and 3 s may have been too long in Experiment 1, where detection accuracy was 81.92%. For Experiment 2, the display duration was reduced to test performance with an increase in the difficulty of the task. The results indicated that even at 2 s, the echocardiographers were accurate in detecting the presence of abnormal dysfunction (82.82%). To establish a baseline of performance and explore a more “real-world” duration, in Experiment 3 we presented two durations (brief and extended) to a group of echocardiographers. Even at a duration of 1 s, D prime for detection was above chance and accuracy on detection remained high (88.43%). Importantly, the raw data demonstrate that performance was comparable with our “real-world” movie duration of 10 s.

For the echocardiographers, dysfunction categorization accuracy was above chance levels (Experiments 1 and 2), but not at ceiling (Experiment 1: 61.84%; Experiment 2: 63.12%; Experiment 3 [1 s]: 58.88%, [10 s]: 61.75%). These results may reflect task demands, where, retaining in short-term memory, the categorization information through an initial detection response and subsequent response screen may have proven cognitively demanding. At a duration of 2–3 s, only 2–3 cardiac cycles were observed for each case and at a duration of 1 s, only 1–2 cycles. In Experiment 3, when seven echocardiographers viewed the longer 10s presentation duration and thus an increase in the number of cardiac cycles, we observe only a slightly higher accuracy in the raw data for categorization. This suggests that the results from Experiments 1 and 2 are indicative of clinical practice.

Alternatively, the results may reflect actual practice and indicate an area of deficiency. Indeed, in a pediatric cardiac setting, discrepant echocardiogram diagnoses accounted for 15% of overall diagnostic error (Benavidez et al. 2014). In a typical scanning scenario, the echocardiographer may acquire images and generate a preliminary report, which (unless deemed urgent) is reviewed and reported by a physician at a later time or date. Information about any minor errors regarding LV dysfunction in the echocardiographers’ report (corrected by the physician) may never return to the echocardiographer. Due to the lack of feedback, echocardiographers may often continue to report dysfunction incorrectly, unaware of their minor error(s). These findings may also be an argument for the quantitative assessment of the LV, which, although would take longer, may reduce categorization variability.

In diagnostic medicine, although high levels of performance are expected, practitioners rarely receive feedback about their performance, especially on a case-by-case basis. This is potentially problematic as a practitioner may have many years of experience, yet be making repetitive errors. Diagnostic skills are learned early in training but once competence is reached, instructional support is removed. Studies in the driving domain have shown that the consequences for errors are most serious in the period immediately after training in the early stages of learning (Kim et al. 1995). At this stage, practitioners are vulnerable to errors and have been considered as having a “license to learn” (Beanland et al. 2013).

During the early stages of learning, rules of thumb are acquired that form the basis of cue associations in memory between features /objects and events (Loveday and Wiggins 2014; Wiggins 2014, 2015; Wiggins et al. 2014). For example, an echocardiographer in the early stages of learning might be taught to associate abnormal heart muscle movement with a disruption to the heart’s blood supply. However, there are situations where these “rules of thumb” fail to hold true and/or lack the precision necessary to formulate a sufficiently meaningful assessment across a range of situations (Drexler et al. 2014). The consequent demand for improved performance provides the impetus for learners to refine, recategorize, or reconstruct cue associations that may have been acquired during the early stages of learning (Palmeri 1999). A lack of ongoing instructional support to help facilitate this transitional period may increase the vulnerability to errors and explain some of the errors in the current study.

Across three experiments, proportionally more errors occurred for the mild and moderate categories, suggesting that this discrimination was particularly difficult. Within the incorrect categorization trials, most echocardiographers responded with “severe.” This provides evidence to suggest that, when faced with uncertainty, the participants adopted a more liberal criterion and chose the more serious level of dysfunction. These findings can be regarded as false positives and, in practice, might mean further unnecessary tests and procedures. For the false alarm responses, the majority categorized dysfunction as “mild,” suggesting that they were able to rule out the moderate and severe cases. Another possibility is that some of the participants may not have been familiar with forming such precise distinctions in their routine practice (mild/moderate/severe). However, for Experiment 2, when explicitly asked about dysfunction labels (post experiment), the majority reported using mild, moderate, or severe categories.

In Experiments 1 and 2, self-reported experience was not related to performance on the detection task. However, in Experiment 1, for the categorization task the Bayes Factor suggests that there was anecdotal evidence for the alternate hypothesis: those with more experience were more accurate in categorizing abnormal ventricular function. This finding was not replicated in Experiment 2 and may reflect variability due to the different sample sizes for each experiment. Moreover, this effect was not strong and there may be other factors at play such as learned strategies (Williams and Drew 2019).

Although years of experience is often regarded as an indicator of expertise, this factor is difficult to measure as it represents overlapping variables that incorporate a number of dimensions which increase the variability in performance (Heilman and Stopeck 1985), such as individual differences. Studies in radiology have shown that experience bares little relationship with visual tasks such as spatial attention cueing (Carrigan et al. 2019a, 2019b), visual search strategies (Williams and Drew 2019), or nodule detection (Sunday et al. 2018). Medical image perception researchers are finding that it is difficult to tease apart experience and learned strategies (Williams and Drew 2019), suggesting that there are other several other factors contributing to the development of expertise. The rate at which skills are acquired is determined by the quality of the experience, opportunities for feedback, and inherent capabilities such as motivation (Ackerman 2014). Echocardiography is predominately a visual task. Therefore, it is plausible that, aside from experience, individual differences in factors such as cue utilization and visual recognition can be identified. Discovering this information may then be applied to assist with targeted training, skill development, and employment selection.

Individual differences in visual perception is another avenue for future research in echocardiography. In many domains, it remains unclear how much variation in performance is due to training and what proportion is contributed by other factors such as general perceptual ability. One way to study this involves using a task which measures domain-general visual recognition ability, such as the Novel Object Memory Test (NOMT) (Richler et al. 2017). A recent study with radiologists showed that experience alone accounted for approximately 50% of the variance in diagnostic performance. When controlling for experience, fluid intelligence and performance on the NOMT accounted for an additional 15% (Sunday et al. 2018). Little is known about domain general perceptual expertise in medical imaging, so this opens up several future experiments which include investigating an inherent level of perceptual expertise. Another future study quantifying dynamic visual search using eye-tracking methodology to examine the precise features within the images that echocardiographers attend to when making decisions, would also be beneficial.

To our knowledge, this study is the first to investigate the early visual processing of echocardiographers by presenting a domain specific visual search task. We demonstrated that, although detection of an obvious abnormality was high, performance distinguishing the degree of abnormality was less so. These results were not strongly related to experience suggesting that there are other factors such as low levels of ongoing support and individual differences affecting the outcomes. This research advances the understanding of the visual and cognitive processes of echocardiographers and provides groundwork for future studies.

## Data Availability

Data can be made available by contacting the corresponding author, Dr. Ann Carrigan, ann.carrigan@mq.edu.au
